# TGFβ Signaling in Myeloid Cells Promotes Lung and Liver Metastasis Through Different Mechanisms

**DOI:** 10.3389/fonc.2021.765151

**Published:** 2021-11-18

**Authors:** Cristina Stefanescu, Merel Van Gogh, Marko Roblek, Mathias Heikenwalder, Lubor Borsig

**Affiliations:** ^1^ Institute of Physiology, University of Zurich, Zurich, Switzerland; ^2^ Institute of Science and Technology (IST) Austria, Klosterneuburg, Austria; ^3^ Division of Chronic Inflammation and Cancer, German Cancer Research Center (DKFZ), Heidelberg, Germany; ^4^ Comprehensive Cancer Center Zurich, Zurich, Switzerland

**Keywords:** tumor microenvironment, metastasis, TGFβ, mouse model, lung, liver, myeloid cells

## Abstract

TGFβ overexpression is commonly detected in cancer patients and correlates with poor prognosis and metastasis. Cancer progression is often associated with an enhanced recruitment of myeloid-derived cells to the tumor microenvironment. Here we show that functional TGFβ-signaling in myeloid cells is required for metastasis to the lungs and the liver. Myeloid-specific deletion of Tgfbr2 resulted in reduced spontaneous lung metastasis, which was associated with a reduction of proinflammatory cytokines in the metastatic microenvironment. Notably, CD8^+^ T cell depletion in myeloid-specific *Tgfbr2*-deficient mice rescued lung metastasis. Myeloid-specific *Tgfbr2*-deficiency resulted in reduced liver metastasis with an almost complete absence of myeloid cells within metastatic foci. On contrary, an accumulation of Tgfβ-responsive myeloid cells was associated with an increased recruitment of monocytes and granulocytes and higher proinflammatory cytokine levels in control mice. Monocytic cells isolated from metastatic livers of *Tgfbr2*-deficient mice showed increased polarization towards the M1 phenotype, Tnfα and Il-1β expression, reduced levels of M2 markers and reduced production of chemokines responsible for myeloid-cell recruitment. No significant differences in Tgfβ levels were observed at metastatic sites of any model. These data demonstrate that Tgfβ signaling in monocytic myeloid cells suppresses CD8^+^ T cell activity during lung metastasis, while these cells actively contribute to tumor growth during liver metastasis. Thus, myeloid cells modulate metastasis through different mechanisms in a tissue-specific manner.

## Introduction

The main cause of cancer-related fatalities in patients is metastasis, a multistep process enabling tumor cells to spread through blood circulation and to form metastasis in distant organs. Stromal cells such as fibroblasts, endothelial and immune cells are integral parts of both primary tumor and metastatic lesions (1, 2). Tumor cells together with stromal cells produce cytokines that orchestrate the formation of a tumor microenvironment, which promotes cancer progression. The TGFβ family of cytokines are directly linked to tumorigenesis, where they actively promote tumorigenesis through a modulation of the tumor microenvironment during metastasis (3–5).

TGFβ cytokines are involved in a wide range of biological processes, such as cell proliferation, differentiation, wound healing, and immune cell regulation (3). TGFβ-signaling is initiated by binding to TGFβ receptor I (TGFβRI), which recruits and phosphorylates TGFβ receptor II (TGFβRII), and results in activation of: the canonical pathway, involving SMAD proteins regulating a wide range of genes; and the non-canonical pathway that includes MAPK and PI3K signaling (4). In the context of cancer, TGFβ1 can act both as a tumor suppressor, in the early stages of cancer, and as a tumor promoter, in the later stages of tumor progression. In advanced cancers, however; TGFβ expression and activation strongly correlate with poor prognosis due to metastasis (4, 6).

TGFβ is an immunosuppressive cytokine produced by tumor cells and immune cells that polarizes both the innate and the adaptive immune system (7) and represents a primary mechanism of immune evasion (8). TGFβ inhibits NK cell function by decreasing cytokine production and in dendritic cells downregulates the expression of MHCII and co-stimulatory molecules. Within the tumor microenvironment, TGFβ signaling promotes immune suppression of macrophages and neutrophils, which results in alternatively polarized phenotypes of M2 and N2, respectively. In the lymphoid compartment, TGFβ dampens the activity of CD4^+^ T helper and of CD8^+^ cytotoxic T cells and promotes the activity of regulatory T (Treg) cells (7).

Myeloid cells promote immune suppression and formation of a metastatic niche and are abundantly present in cancer patients both as circulating cells in the blood and in tumor tissues (9–12). Systemic expansion of immature myeloid cells during cancer progression can be divided into two subpopulations of granulocytic (CD11b^+^/Ly6G^+^) and monocytic (CD11b^+^/Ly6C^hi^) myeloid-derived suppressor cells (9). In the tumor microenvironment TGFβ is an important factor promoting immune-suppressive polarization of myeloid-derived cells. Breast cancer metastasis to the lungs was dependent on myeloid cell recruitment and formation of pro-tumorigenic microenvironment (13). Inhibition of Tgfβ in tumor bearing-mice resulted in increased infiltration of cytotoxic CD11b^+^/Ly6G^+^-derived neutrophils and reduced tumor growth (14). Initial studies in mice with *Tgfbr2*-deficiency in myeloid cells showed reduced tumor growth, which was associated with an increased anti-tumor polarization of macrophages (15). Further studies in *Tgfbr2*-myeloid cell deficient mice showed reduced lung metastasis in 4T1 mammary tumor model (16, 17), which was ascribed to enhanced presence of cytotoxic CD8^+^ T cells (16). Tumor-associated myeloid cells express CCL9 chemokine, which is upregulated in a TGFβ-dependent manner (17). The overexpression of CCL9 in *Tgfbr2*-deficient myeloid cells enabled metastasis. In a recent study, the CXCL1-CXCR2 axis was identified to mediate myeloid cell recruitment to the pre-metastatic liver (10). However, the contributing effects of TGFβ signaling during liver metastasis remain unclear.

The current study investigates and compares the tissue-specific role of TGFβ signaling in myeloid cells and its influence on the metastatic microenvironment in lung and liver metastasis using a LysMCre/*Tgfbr2^fl/fl^* mouse model.

## Materials and Methods

### Cell Culture

MC-38GFP murine colon carcinoma cell line (18), LLC1.1 Lewis lung carcinoma, 3LL lung carcinoma, and B16-BL6 were grown in DMEM high glucose medium (Life Technologies) containing 10% fetal bovine serum (FBS) as described previously (19).

### Mice

LysMCre/*Tgfbr2^fl/fl^* mouse in a C57BL/6J background was obtained from M. Heikewälder (University of Zurich). The mouse strain *ROSA-STOP^fl/fl^-dTomato* was obtained from The Jackson Laboratory. All mice were in the C57BL76J background. Both males and female animals in the age of 7-10 weeks of age were used in all experiments. All animal experiments were performed according to the guidelines of the Swiss Animal Protection Law, and approved by the Veterinary Office of Kanton Zurich.

### Metastasis Models

*Spontaneous lung metastasis.* Lewis lung carcinoma cells LLC1.1 (300,000 cells) were subcutaneously (s.c.) injected into a right flank of a mouse. The primary tumor was removed after 18 days and the extent of lung metastasis was evaluated two weeks after tumor removal. *Experimental liver metastasis.* MC-38GFP colon cancer cells (300,000 cells) were injected into the spleen of an anesthetized mouse. Splenectomy was performed three minutes after the tumor cell injection. Mice were terminated on day 28 and liver metastasis was evaluated. *Experimental lung metastasis.* MC-38GFP (300,000 cells) were intravenously injected and mice were terminated 28 days after the injection. 3LL and B16-BL6 cells (150,000 cells) were intravenously injected and lungs analyzed on day 14 (19). Surface metastasis were counted in perfused lung and liver tissues.

### Immunohistochemistry

Tissues paraffin sections (3 μm) were stained with hematoxylin/eosin or the anti-GFP antibody (Fitzgerald Industries Int.) Ki67 (NeoMarkers) and cleaved caspase-3 (BD). Quantification of tumor cells in seven sections of lungs separated by 20 μm was performed. Staining was performed on a NEXES immunohistochemistry robot using an IVIEW DAB Detection Kit (Ventana Instruments, Switzerland). Images were digitalized on Zeiss MiraxMidi Slide Scanner and analyzed with Panoramic Viewer (3DHISTECH). For cryosections, tissues were fixed in 3% paraformaldehyde for 1 hour at 4°C, incubated in 20% sucrose overnight at 4°C, embedded in an OCT compound and snap-frozen in a bath containing methylbutane and dry ice. Cryosections (10 μm) were blocked with phosphate buffer saline (PBS) containing 5% FCS and 5% rat serum, and incubated with AlexaFluor 647-coupled rat-anti-F4/80 antibody (Clone BM8, Biolegend). Slides were counterstained with DAPI and embedded in Prolong and evaluated on Leica Thunder Microscope.

### Flow Cytometry Analysis

Mice were perfused with PBS and dissected tissues were minced and digested with Collagenase A and D (1 mg/mL, both from Roche), in 2 mL of RPMI medium containing 3% BSA for 1 hour at 37°C. The cell suspension was prepared by passing the homogenate five times through 18G needle followed by a 100μm cell strainer. In case of liver, the cell suspension was centrifuged at 500 rpm for one minute to separate the parenchyma. After centrifugation of the single cell suspension, erythrocytes lysed with ACK buffer and cells were passed through a 70μm cell strainer. The pellet was resuspended in a FACS buffer (PBS/2% FBS/5mM EDTA) and stained with Zombie Fixable Viability Kit (Biolegend), followed by incubation with anti-mouse CD16/32 mAb for 10 min on ice. Single cell suspensions were stained with the following antibodies: anti-CD45 (clone 30-F11), anti-CD11b (clone M1/70), anti-F4/80 (clone BM8), anti-Ly6G (clone 1A8), anti-Ly6C (clone HK1.4), anti-MHCII (clone M5/114.15.2), anti-CD31 (clone 390), CD4 (clone GK1.5), anti-CD8 (clone 53-6.7) and anti-PD1 (clone 29F.1A12) (all from Biolegend) for 30 min on ice. Data was acquired on a FACSCanto II (BD Biosciences), during cell quantification together with CountBright absolute counting beads (Life Technologies) and analyzed using Flow Jo software v. 7.6.5 (Tree Star). Samples for CD8 cell activation assessment were fixed and permeabilized using FoxP3/Transcription Factor Staining Buffer Set (eBioscience). Subsequently samples were stained for intra-cellular markers with the following antibodies: anti-Granzyme B (clone GB11) and anti-Perforin (clone S16009A) from Biolegend. CD8 cell activation was analyzed using the Cytek Aurora 5L – Spectral Analyzer from the Cytometry Facility at the University of Zurich.

### Cell Sorting and Quantitative PCR

Tissues were processed the same way as described for flow cytometry analysis. Individual cell populations were sorted using a BD Aria III in FACS buffer at 4°C using a 100 μm nozzle. From at least 100’000 sorted cells RNA was isolated using the RNA Easy Plus Mini kit (Qiagen) and cDNA was prepared using 250 ng of RNA using Omniscript RT kit (Qiagen) according to the manufacturer’s instructions. Real-time PCR was performed with KAPA Sybr FAST Universal (Sigma) using intron-spanning primers (**Supplementary Table S1**) and analyzed in a CFX96 Touch Real-Time PCR Detection System (Biorad). GAPDH was used as an internal control.

### Fluorescent Beads Uptake Assay

Red fluorescence beads (Sigma) were diluted 1:20 in PBS and 100 μl was intravenously injected through a tail-vein per mouse. Mice were terminated after 9 hours, blood and organs were collected and processed as described in flow cytometry analysis.

### Reactive Oxygen Species (ROS) Determination

Tumor bearing mice (subcutaneous LLC1.1 tumor) were anesthetized, the lungs perfused with PBS and through heart perfused with pre-warmed 5 μM CellROX^®^ Oxidative Stress Green Reagent (Invitrogen) and the vessels clipped. After 30 min of incubation, the lungs were again perfused with PBS, digested with collagenase and single cell suspension stained with anti-Ly6G antibody and the ROS signal was quantified by flow cytometry.

### Tumor Cell *In Vivo* Seeding Assay

MC-38-luciferase expressing cells (300,000) were injected through spleen in mice and at indicated time points, mice were anesthetized, subcutaneously injected with D-luciferin firefly (150 μg/g mouse) and after 8 min terminated. Mice were perfused with PBS and the luciferase activity in the liver was determined using IVIS imaging. After the intravenous injection of 300,000 MC-38GFP cells mice were terminated at indicated time points; mice perfused with PBS and lungs processed for histological analysis.

### Antibody Depletion Experiments

Depletion of CD8^+^ T cells in a lung metastasis model using LLC1.1 cells was performed with 10 μg anti-CD8 antibody (clone 2.43; BioXCell) i.v. injected one day prior to primary tumor removal. Mice received another 15 μg of the antibody by i.v. injection two days after the tumor removal. Control mice received the isotype control antibody (clone 2A3, BioXCell) in parallel. In the liver metastasis model, mice received 10 μg Ab injection 1 day before tumor cell injection and the second treatment with 15 μg Ab two days after tumor cell injection.

Neutrophil depletion was achieved by intraperitoneal injection (i.p.) of anti-Ly6G antibody (clone 1A8; BioXCell). Initial injection of 500 μg/mouse was performed 24 hours prior to intrasplenic injection of MC-38GFP cells. Mice were treated three times weekly with 100 μg i.p. until the termination at day 27. Control animals were injected with isotype-control Ab (clone 2A3, BioXCell) in parallel.

### Cytokine Analysis

Snap-frozen tissues were homogenized using Polytron^®^ in lysis buffer (FACS buffer containing 1x complete EDTA free proteinase inhibitor cocktail from Roche). After 15 min centrifugation at 14’000 rpm at 4°C, was determined the protein concentration in the supernatant was determined with BCA Protein Assay (Thermo Fischer). Cytokine concentrations were measured in a total of 200-500 μg of tissue lysate using the ProcartaPlex 23 Plex and the LegendPlex Mouse Proinflammatory Chemokine panel (Thermo Fischer) following the manufacturer’s instructions. TGFβ1 was determined using the TGFβ Platinum Elisa (Thermo Fisher), where samples were activated with 1N HCl for 10 min at RT in a ratio of sample to HCl 5:1. The reaction was neutralized using 1 part of 1N NaOH/0.5M HEPES. Sorted tdT^+^ Ly6C^hi^ monocytes (20’000 cells/well) from livers of mice at day 14-post-intrasplenic injection of MC-38GFP cells were stimulated overnight (14 hrs) with PMA (20 ng/ml), ionomycin (1μg/ml) and LPS (1 μg/ml). Cytokine production was detected in the supernatant after 14 h stimulation as described above.

## Results

### Myeloid-Specific Deletion of Tgfbr2 Attenuates Lung and Liver Metastasis

Myeloid cells are the dominant population of immune cells associated with metastatic progression (20–22); and TGFβ is an immunosuppressive cytokine family that promotes metastases through modulations of the tumor microenvironment (7). To assess the role of Tgfβ-signaling in myeloid cells during lung and liver metastasis, we generated a mouse with myeloid cell-specific deletion of TGFβ receptor II (LysMCre^+^/*Tgfbr2^fl/fl^* mouse, hereafter TR2^my^KO) by crossing the *Tgfbr2^fl/fl^* mouse with a mouse expressing Cre under the control of the Lysozyme promoter (LysMCre). LysMCre^neg^/*Tgfbr2^fl/fl^* (hereafter Ctrl) served as a control.

**Figure 1:**
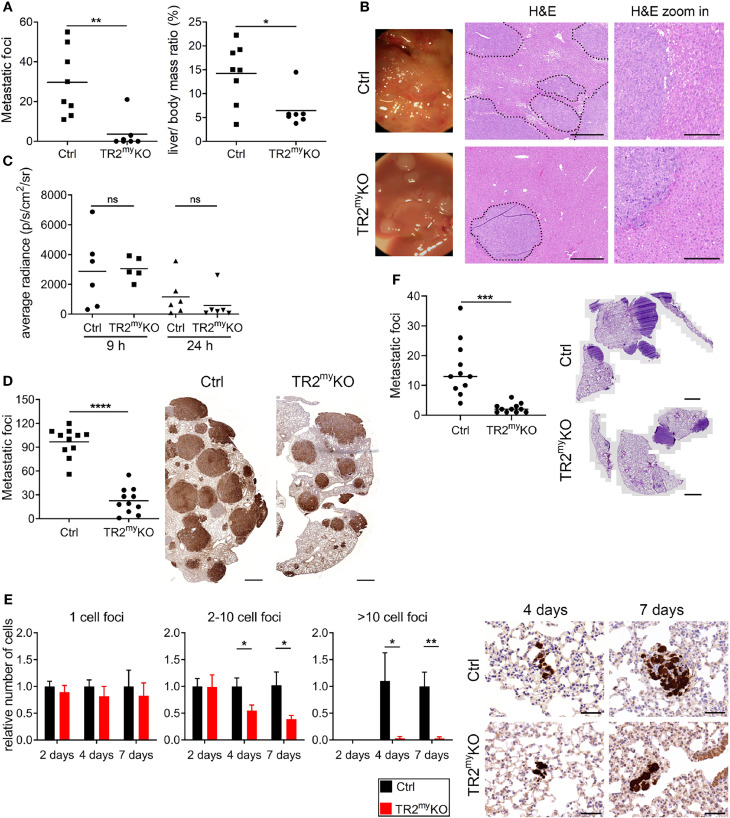
Attenuation of liver and lung metastases in mice with myeloid cell-deletion of Tgfbr2. **(A)** Macroscopic quantification of liver metastatic foci and the ratio of liver weight to the total body mass (%) in TR2^my^KO and control (Ctrl) mice 28 days after the intraspenic injection of MC-38GFP cells. **(B)** Representative macroscopic liver images and hematoxylin/eosin (H&E) stained liver sections indicating liver metastasis. Dotted line represents the edge of metastatic foci (middle panel). Bar = 500 μm; bar zoom-in = 200 μm. **(C)** Liver seeding of MC-38 luciferase-expressing cells determined at 9 and 24 hours post-intrasplenic injection of MC-38 luciferase cells using quantification of luciferase signal in the perfused liver. **(D)** Macroscopic quantification of lung metastasis in TR2^my^KO compared to control (ctrl) mice 30 days after intravenous injection of MC-38GFP cells. Representative images (right panel) of pulmonary metastasis stained with anti-GFP antibodies (brown). Bar = 1 mm. **(E)** Quantification of GFP^+^ cells in lung sections at 2, 4 and 7 days post-tumor cell injection as tumor cell clusters divided into: 1 cell, 2-10 cell foci and >10 cell foci. Representative images of lung sections from day 4 and 7 stained for GFP (brown). n = 4-6 mice per time point and genotype. Bar = 50 μm. **(F)** Spontaneous lung metastasis of LLC1.1 cells subcutaneously injected in TR2^myelo^KO and Ctrl mice terminated at days 32. Representative H&E stained lung sections of respective genotype. Bar = 1 mm. For panels **(A–D, F)** each dot represents a mouse. Statistical significance was assessed using Mann-Whitney t-test: ns, not significant; *p < 0.05; **p < 0.01; ***p < 0.001.

First, we tested the role of myeloid cell-derived Tgfβ-signaling during liver metastasis. We used an experimental liver metastasis model, where MC-38 murine colon carcinoma cells stably expressing GFP (MC-38GFP) were injected into the spleen. Significant reduction of metastatic foci and liver-body mass ratio was observed in TR2^my^KO mice when compared to Ctrl mice (**Figure 1A**). The size of liver metastatic lesions was also significantly reduced in TR2^my^KO mice (**Figure 1B**). To test whether myeloid cells affect tumor cell seeding to the liver in a Tgfβ-dependent manner, we intrasplenically injected MC-38-luciferase expressing cells and analyzed livers 9 and 24 hours post-tumor cell-injection using bioluminescence imaging (**Figure 1C**). No difference in tumor cell detection in the liver was observed between TR2^my^KO and Ctrl mice, indicating that Tgfβ-signaling pathway in myeloid cells is not affecting initial tissue colonization.

Second, we tested whether Tgfβ-signaling deficiency in myeloid cells affects lung metastasis. Myeloid cells were previously shown to promote metastatic seeding to the lungs (22–24). Using three different cell lines, MC-38, Lewis lung carcinoma 3LL and B16-Bl6 melanoma in experimental lung metastasis models, we observed reduced metastasis in TR2^my^KO mice (**Figure 1D** and **Supplementary Figures 1A, B**). To assess the effect of myeloid Tgfβ-deficiency on lung metastasis, we analyzed tissues sections two, four and seven days post-tumor cell injection. While the number of single tumor cells was similar in TR2^myelo^KO and Ctrl mice at every time point, we observed a significant reduction of small clusters (2-10 tumor cells) and large cluster (more than 10 tumor cells) at day 4 and day 7 post-tumor cell challenge in TR2^my^KO mice (**Figure 1E**). These findings indicate that the metastatic outgrowth in TR2^my^KO mice has been greatly hampered (2-10 cells foci) or impaired (>10 cells foci). Next, we used the spontaneous lung metastasis model, where mice were subcutaneously (s.c.) injected with Lewis lung carcinoma (LLC1.1) cells. At day 18 the primary tumor was surgically removed and after another 14 days lung metastasis was analyzed. While no difference in the primary tumor growth between TR2^my^KO and Ctrl mice was observed (**Supplementary Figure 1C**), a significant reduction of lung metastasis was detected in TR2^my^KO mice (**Figure 1F**).

### Increased Presence of Tgfβ-Responsive Myeloid Cells During Liver Metastasis

**Figure 2:**
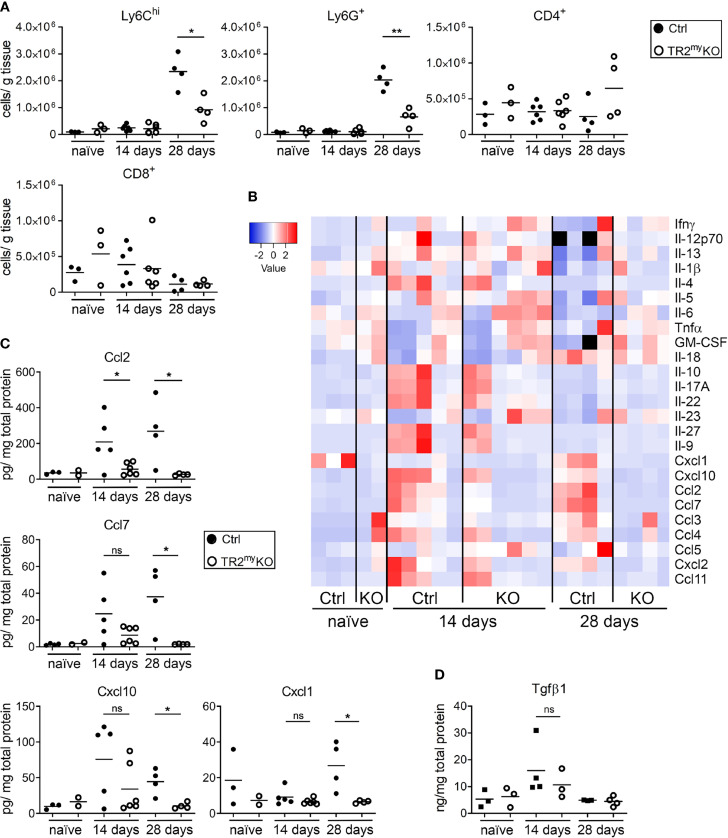
Analysis of liver metastasis in Tgfbr2-myeloid cell-deficient mice. **(A)** Flow cytometry analysis of Ly6C^hi^, Ly6G^+^ myeloid-derived cells and CD4^+^ and CD8^+^ lymphocytes in livers of TR2^my^KO and Ctrl mice upon intrasplenic injection of MC-38GFP cells that were terminated at day 14 or 28, respectively; and compared to naïve mice. **(B)** Determination of cytokine amounts in the perfused liver homogenates of TR2^my^KO and Ctrl mice at day 14 and 28 post-intrasplenic injection with MC-38GFP cells. **(C)** Quantification of selected cytokines amounts with significant changes among TR2^my^KO and Ctrl mice samples. **(D)** Tgfβ1 levels in the liver of mice after intrasplenic injection of MC-38GFP cells 14 and 28 days post-injection when compared to naïve mice without any tumor cell injection. Statistical significance was assessed using Mann-Whitney test: ns, not significant; *p < 0.05; **p < 0.01.

To understand the role of myeloid cell Tgfβ-signaling during liver metastasis, we analyzed these cells in metastatic livers at two different time points after tumor cell injection: 14 days – microscopic metastasis, 28 days – macroscopic metastasis, and compared the changes to naïve mice. We observed increased numbers of inflammatory monocytes (Ly6C^hi^) and neutrophils (Ly6G^+^) in Ctrl mice, which were significantly reduced in TR2^my^KO at day 28 (**Figure 2A**). There were no changes in lymphoid CD4^+^ or CD8^+^ cells at any time point. Next, we analyzed cytokine levels in metastatic livers (**Figure 2B**). Increased levels of Ccl2, Ccl7, Cxcl1, and Cxcl10 chemokines both at day 14 and at day 28 in Ctrl mice correlated well with respective increased cytokine levels (**Figure 2C**). Both lower levels of cytokines and reduced myeloid cell number were detected in TR2^my^KO mice. Increased amounts of Tgfβ1 cytokine were detected in metastatic livers at day 14, irrespective of mouse genotype, which decreased to levels observed in naïve mice at day 28 (**Figure 2D**).

**Figure 3:**
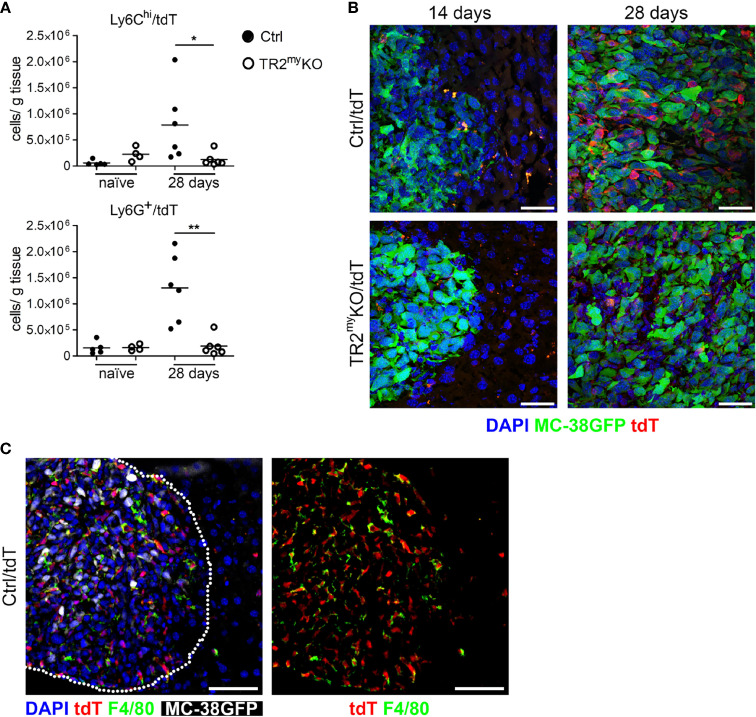
Reduced myeloid cell recruitment to liver metastatic foci in *Tgfbr2*-myeloid cell-deficient mice. Analysis of tdTomato reporter expression in TR2^my^KO (LysMCre^+^/*Tgfbr2^fl/fl^*/tdT^+^) and Ctrl mice (LysMCre^+^/*Tgfbr2^wt^*/tdT^+^). **(A)** Flow cytometry analysis of tdT-positive Ly6C^hi^ and Ly6G^+^ myeloid cells in perfused livers from TR2^my^KO and Ctrl mice at day 28 post-intrasplenic tumor cell injection compared to non-injected mice (naïve). **(B)** Representative images of metastatic liver foci at day 14 and 28 post-tumor cell injection in TR2^my^KO and Ctrl mice showing tumor cells (green), and myeloid cells (red) and counterstained with DAPI (blue). Bar = 30 μm. **(C)** Immunofluorescence analysis of F4/80-stained macrophages in livers 28 days after MC-38GFP splenic injection in Ctrl mice. Dotted line represents the edge of a metastatic focus (left panel); tdTomato channel and F4/80 staining (right panel) Bar = 50 μm. Statistical significance was assessed using Mann-Whitney test: *p < 0.05; **p < 0.01.

Next, we aimed to detect myeloid cells in liver metastatic lesions. We prepared a mouse by breeding of LysMCre^+^/*Tgfbr2^fl/fl^* strain into a transgenic mouse, Ai14, carrying loxP-flanked STOP cassette followed by tdTomato inserted into the ROSA26 locus. We analyzed myeloid cells from naïve LysMCre^+^/*Tgfbr2^fl/fl^*/tdT^+^ mice (TR2^my^KO/tdT) and LysMCre/*Tgfbr2^wt^*/tdT^+^, wild-type allele (Ctrl/tdT) mice, and detected over 90% tdT^+^ Ly6G^+^ and over 30% tdT^+^ of Ly6C^hi^ cells in liver, lungs and spleen (**Supplementary Figure 2**). When MC-38GFP tumor cells were injected intrasplenically into TR2^my^KO/tdT and Ctrl/tdT mice, we observed increased numbers of both Ly6C^hi^ and Ly6G^+^ cells in Ctrl/tdT mice by flow cytometry, while in TR2^my^KO/tdT mice these myeloid cells were hardly present in the metastatic lesions (**Figure 3A**). These results confirm our previous data (**Figure 2A**). The histological analysis of liver sections at day 14 and 28 days post-tumor cell injection substantiated the finding of a pronounced reduction of tdT-positive cells within metastatic foci in TR2^my^KO/tdT when compared to Ctrl/tdT mice (**Figure 3B**). Furthermore, large proportion of tdT-positive cells were positive for an F4/80 antigen (**Figure 3C**), indicating differentiation of myeloid-derived cells into macrophages. Histological analysis of metastatic livers for proliferating cells (Ki67^+^ cells) and for apoptotic cells (cleave-caspase3^+^ cells) revealed no obvious differences between Ctrl and TR2^my^KO mice (**Supplementary Figure 3**). These data show a reduced presence of Tgfβ-responsive myeloid cells inside the growing metastatic foci, which correlated with decreased amounts of chemokines in the metastatic livers in TR2^my^KO mice.

### Tgfβ-Mediated Polarization of Monocytic Myeloid Cells Promotes Liver Metastasis

**Figure 4:**
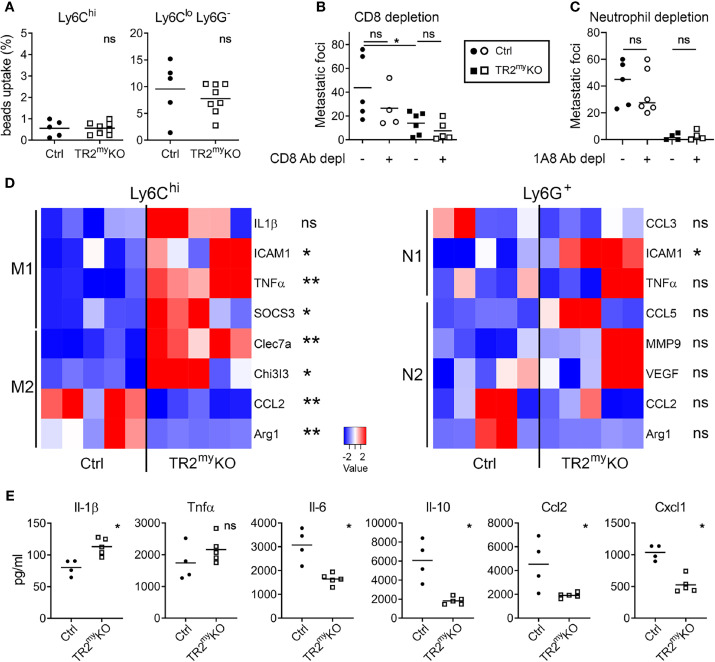
Altered polarization of *Tgfbr2*-deficient monocytic cells resulted in reduced liver metastasis. Characterization of myeloid cells from TR2^my^KO and Ctrl mice at day 14 post-intrasplenic injection of MC-38GFP cells. **(A)** The phagocytic capacity of Ly6C^hi^ monocytes and Ly6C^lo^/Ly6G^-^ macrophages isolated from metastatic livers 9 hours after the intravenous injection of fluorescent beads. **(B)** Liver metastasis of MC-38GFP cells upon anti-CD8 antibody or isotype control depletion in TR2^my^KO and Ctrl mice. Mice were terminated at day 28 and liver metastasis quantified. **(C)** Liver metastasis of MC-38GFP cells upon anti-Ly6G (1A8) antibody or isotype control depletion in TR2^my^KO and Ctrl mice. Mice were terminated at day 28, and liver metastasis quantified. **(D)** Gene expression analysis of Ly6C^hi^/tdT^+^ monocytes and Ly6G^+^/tdT^+^ neutrophils sorted from livers of tdT-reporter TR2^my^KO and Ctrl mice at day 14 post-tumor cell injection performed by qPCR. A panel of genes associated with M1 and M2 macrophage polarization and N1 and N2 neutrophil polarization was analyzed. **(E)** Analysis of cytokine production by tdT^+^ sorted Ly6C^hi^ monocytes from livers of TR2^my^KO and Ctrl mice 14 days post-intrasplenic injection of MC-38GFP cells. Cells were stimulated overnight and secreted cytokines were measured in supernatants. Statistical significance was assessed using Mann-Whitney test: ns, not significant; *p < 0.05; **p < 0.01.

To assess how Tgfβ-responsive myeloid cells promote liver metastasis, we first analyzed myeloid cells from metastatic livers of TR2^my^KO and Ctrl mice. First, the phagocytic activity of myeloid-derived (CD11b^+^) cells was tested. We analyzed Ly6C^+^ monocytes and Ly6G^lo^/Ly6C^-^ macrophages isolated from livers of mice intrasplenically injected with MC-38 cells at day 14, during early metastasis. No differences in phagocytic capacity of monocytic cells were observed between the respective mouse genotypes (**Figure 4A**). A recent study using a murine breast cancer model with 4T1 tumor cells in LysMCre^+^/Tgfbr2^fl/fl^ mice showed that CD8^+^ T cells control lung metastasis (16). Thus, we depleted CD8^+^ T cells to test whether myeloid cells modulate CD8^+^ T cells and thereby promote liver metastasis. While efficient depletion of CD8^+^ T cells in the blood liver and lungs has been achieved (**Supplementary Figure 4A**), no effect on liver metastasis has been observed (**Figure 4B**). This finding points to a different mechanism of Tgfβ-stimulated pro-metastatic myeloid cell involvement during liver metastases. To determine the subpopulation of myeloid cells involved in liver metastasis, we depleted Ly6G^+^ cells during experimental liver metastasis. Depletion of neutrophils showed no difference in liver metastasis neither in Ctrl nor in TR2^my^KO mice (**Figure 4C**), suggesting the involvement of myeloid-derived monocytic cells.

The altered polarization of monocytes/macrophages and neutrophils towards M2 and N2 phenotype, respectively, was previously associated with cancer progression (25, 26). Therefore, we sorted Ly6C^hi^ monocytes and Ly6G^+^ neutrophils from metastatic livers of TR2^my^KO and Ctrl mice and analyzed the gene expression associated with M1 and M2 polarization (27) and N1 and N2 polarization (28), respectively. We observed an increased expression in seven out of eight genes in monocytic cells from TR2^my^KO mice, which were mostly M1 genes (**Figure 4D**). Specifically, increased expression of ICAM1, TNFα and SOCS3, and decreased expression of M2 genes, CCL2 and Arg1 was detected in Ly6C^hi^ cells from TR2^my^KO mice (**Figure 4D**). However, we observed no significant changes in polarization of Ly6G^+^ cells, while only the ICAM1 was increased in cells from TR2^my^KO mice. To confirm the altered polarization of monocytes, we sorted Ly6C^hi^ cells from metastatic livers and measured cytokine production *in vitro* (**Figure 4E**). Ly6C^hi^ cells from TR2^my^KO mice showed increased production of Il-1β, and partially increased Tnfα levels when compared to monocytes from Ctrl mice. Reduced levels of several cytokines, such as Il-6, Ccl2, Ccl3, Ccl5, Cxcl1 and Cxcl10, but also Il-10 were detected in Ly6C^hi^ cells from TR2^my^KO mice (**Figure 4E** and **Supplementary Figure 4B**). Changes in cytokine production in Ly6C^hi^ cells supports the active role of Tgfβ-signaling in modulation of myeloid-derived cells that facilitates liver metastasis.

### Unchanged Levels of Myeloid Cells During Lung Metastasis

**Figure 5:**
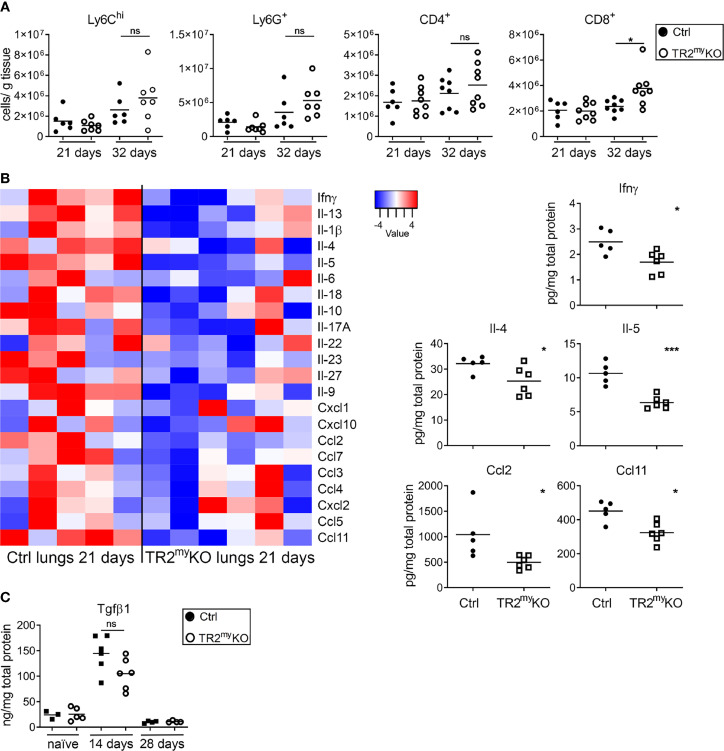
Analysis of spontaneous lung metastasis in *Tgfbr2*-myeloid cell-deficient mice. **(A)** Flow cytometry analysis of the myeloid cells, Ly6C^hi^, Ly6G^+^; and lymphocytes, CD4^+^ and CD8^+^ cells in lungs at day 21 and 32 after the subcutaneous injection of LLC1.1 cells in TR2^myelo^KO mice and Ctrl mice, respectively. **(B)** Determination of cytokine amounts in the perfused lung homogenates of TR2^my^KO and Ctrl mice at day 21. Quantification of selected cytokines amounts (panels) with significant changes among TR2^my^KO and Ctrl mice samples. **(C)** Tgfβ1 levels in the lungs of tumor-bearing mice at day 14 and 28 after subcutaneous injection of LLC1.1 cells, when compared to naïve mice. Statistical significance was assessed using Mann-Whitney test: ns, not significant; *p < 0.05; ***p < 0.001.

We studied how the absence of Tgfb-signaling in myeloid cells reduces lung metastasis. The cellular composition of metastatic lungs in the LLC1.1 spontaneous metastatic model was analyzed. We selected day 21, which is 5 days after the primary tumor removal, as an early metastatic time point; and day 32, as a late metastatic phase. No differences in the presence of Ly6C^hi^ monocytes, Ly6G^+^ granulocytes and CD4^+^ T cells were detected between TR2^my^KO and Ctrl mice at any time point (**Figure 5A**). However, we observed increased numbers of CD8^+^ T cells in the lungs of TR2^my^KO mice at day 32 (**Figure 5A**). Of note, reduced numbers of F4/80^+^ macrophages was observed in the lungs of TR2^my^KO mice at day 21, but not at day 32, when compared to control mice (**Supplementary Figure 5A**). Next, we analyzed cytokine levels in metastatic lungs at day 21 (**Figure 5B**). There was an overall decrease in cytokines in TR2^my^KO mice when compared to Ctrl mice, specifically a significant reduction of Ifnγ, Il-4, Il-5,Ccl2 and Ccl11 was observed. Since tumor-associated myeloid cells can inhibit immune cell functions (20), the observed decrease in Il-4 and Il-5 together with a tendency of Il-10 in TR2^my^KO mice suggests a possible reduced polarization of T cells. The reduced Ccl2 levels detected in TR2^my^KO mice corresponds well with reduced macrophages in the lungs. Finally, we measured the amounts of Tgfβ1 cytokine in the lungs of tumor-bearing mice and compared them with non-injected (naïve) mice (**Figure 5C**). We observed an increase in Tgfβ1 levels in the lungs of tumor-bearing mice at day 14, while at day 28 there was no difference when compared to the lungs of naïve mice. When we analyzed metastatic lungs from TR2^my^KO/tdT and Ctrl/tdT mice at day 32 days after LLC1.1 s.c. injection, we observed no changes in the presence of tdT-positive Ly6C^hi^ and Ly6G^+^ cells between mouse phenotypes as determined by flow cytometry (**Supplementary Figure 5B**).

### Lung Metastasis Are Controlled Through Tgfβ-Myeloid Cell Suppression of CD8^+^ T Cells

**Figure 6:**
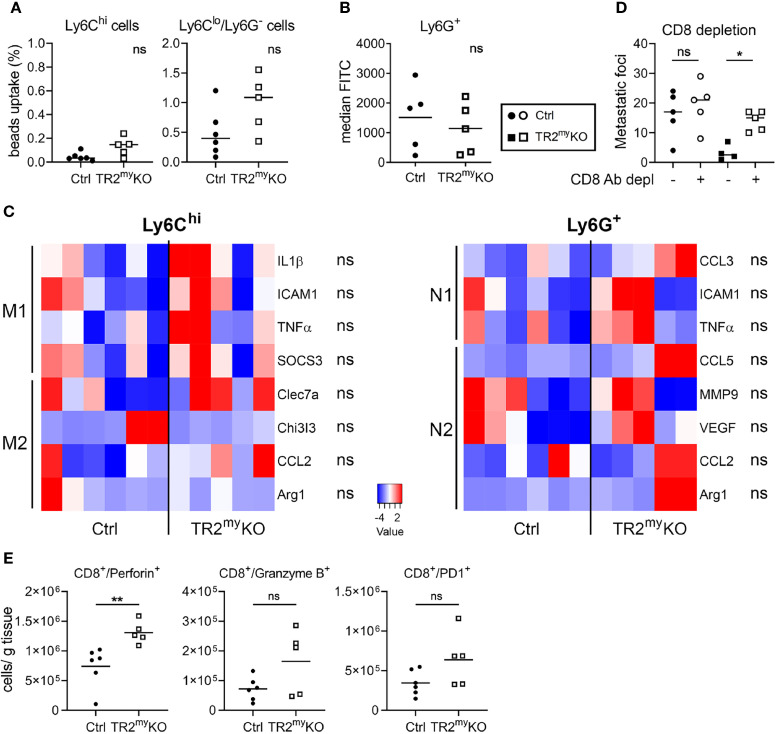
Lung metastasis in *Tgfbr2*-myeloid cell-deficient mice are controlled by CD8 cells. Characterization of myeloid cells from TR2^my^KO and Ctrl mice at day 21 (early metastatic lungs) post-subcutaneous injection of LLC1.1 cells. **(A)** The phagocytic capacity of Ly6C^hi^ monocytes and Ly6C^lo^/Ly6G^-^ macrophages isolated from metastatic lungs 9 hours after the intravenous injection of fluorescent beads. **(B)** Reactive oxygen species (ROS) levels in neutrophils isolated from the lungs of mice perfused with the CellROX^®^ dye *in vivo*. Data are presented in median fluorescent intensity (MFI). **(C)** Gene expression analysis of Ly6C^hi^/tdT^+^ monocytes and Ly6G^+^/tdT^+^ neutrophils sorted from metastasis lungs of tdT-reporter TR2^my^KO and Ctrl mice at day 21 post-tumor cell injection and 5 days after the tumor removal performed by qPCR. A panel of genes associated with M1 and M2 macrophage polarization and N1 and N2 neutrophil polarization was analyzed. **(D)** Spontaneous lung metastasis of LLC1.1 cells upon anti-CD8 antibody or isotype control depletion in TR2^my^KO and Ctrl mice. Mice were terminated at day 32, and lung metastasis quantified. **(E)** Analysis of effector CD8^+^ cells from metastatic lungs at day 32 in a spontaneous lung metastasis model LLC1.1 using flow cytometry analysis for detection of perforin, granzyme B and PD-1. Statistical significance was assessed using Mann-Whitney test: ns, not significant; *p < 0.05; **p < 0.01.

Next, we tested whether attenuated lung metastasis is associated with altered polarization of Tgfβ-signaling deficient myeloid cells, when no major changes in myeloid cell presence in lungs of TR2^my^KO/tdT and Ctrl/tdT mice were detected (**Figure 5A**). First, the phagocytic activity of myeloid-derived (CD11b^+^) cells was tested. We analyzed Ly6C^+^ monocytes and Ly6G^lo^/Ly6C^-^ macrophages isolated from lungs of mice injected with LLC1.1 cells at day 21, during early metastasis. No changes in uptake of beads neither in monocytic cells nor myeloid-derived macrophages isolated from TR2^my^KO and Ctrl mice could be detected (**Figure 6A**). Second, we tested the production of reactive oxygen species (ROS) by myeloid-derived Ly6G^+^ cells, which was similar in both mouse genotypes (**Figure 6B**). These data indicate no functional changes in *Tgfbr2*-deficient myeloid cells in metastatic sites. TGFβ signaling is a critical mediator of immune cell polarization (7). Therefore, we sorted Ly6C^hi^ monocytes and Ly6G^+^ neutrophils from the lungs at day 21, early metastasis, and analyzed their polarization status on a transcriptional level. However, we detected no significant changes in expression of genes linked to respective polarization patterns of myeloid cells (**Figure 6C**). Since we observed enhanced infiltration of lungs metastasis with CD8^+^ T cells in TR2^my^KO mice (**Figure 5A**), we tested their role in control of lung metastasis. Depletion of CD8^+^ T cells in a LLC1.1 model 24 h before the primary tumor removal and during the metastatic phase resulted in increased lung metastasis in TR2^my^KO mice to similar levels as observed in Ctrl mice (**Figure 6D**), indicating that Tgfβ-signaling-deficient myeloid cells control lung metastasis through modulation of CD8^+^ T cells. The analysis of CD8^+^ T cells from metastatic lungs (**Supplementary Figure 6**) showed a significantly increased levels of perforin^+^ CD8^+^ T cells in TR2^my^KO mice when compared to Ctrl mice (**Figure 6E**). However, no major differences in granzyme B expression, nor alteration in PD1 expression has been observed. These data provide evidence that the absence of Tgfβ-signaling in myeloid cells results in increased levels of functional CD8^+^ T cells, which control metastatic outgrowth in the lungs.

## Discussion

Cytokines have a key role in orchestrating the tumor microenvironment both during tumorigenesis and metastasis (29). TGFβ signaling affects primarily the immune compartment of a tumor and stimulates immune suppressive polarization of myeloid-derived cells (3). The abrogation of TGFβ signaling in mammary carcinoma resulted in an increased chemokine production; recruitment of myeloid cells and promotion of lung metastasis (22). On contrary, the absence of Tgfβ signaling in myeloid cells attenuated lung metastasis in a murine mammary tumor model (16, 22). Here we confirmed that functional TGFβ signaling in the myeloid compartment is essential for lung metastasis and provided the first evidence for its involvement also in liver metastasis.

The tumor microenvironment varies between primary tumors and metastatic sites and the composition dynamically changes throughout cancer progression (1, 30, 31). Therefore, we analyzed composition of metastatic lungs and livers for the presence of myeloid cells both in early metastatic phase and in fully developed metastasis. A strong infiltration of tdTomato-positive myeloid cells was observed in metastatic liver foci of Ctrl mice. However, in the very few metastatic foci detected in TR2^my^KO mice, we observed virtually no tdT^+^ cells, indicating that myeloid cells with non-functional TGFβ signaling are unable either to get recruited or to survive in the liver metastatic niche. Closer analysis of liver metastasis, revealed that the majority of tdT^+^ monocytic cells differentiate into macrophages (F4/80^+^ cells).

In a colon cancer mouse model, myeloid cells were shown to promote liver metastasis (10). Tumor cell-triggered increased production of CXCL1 by tumor-associated macrophages resulted in increased myeloid cell recruitment to the premetastatic liver tissue. In agreement with these data, we observed reduced levels of CXCL1, CXCL10 but also CCL2, CCL3 and CCL5 in monocytes (Ly6C^+^ cells) from metastatic livers of TR2^my^KO, with reduced liver metastasis, when compared with Ctrl mice. Importantly, an overall reduction of chemokines responsible for recruitment of myeloid cells was observed in TR2^my^KO mice that correlated with a minimal number of myeloid cells, both monocytic and granulocytic, in liver metastatic foci. Since depletion of neutrophils did not alter liver metastasis, the presence of monocytic myeloid cells is expected to modulate this process. Tgfβ-signaling deficiency in myeloid cells resulted in increased anti-tumorigenic polarization, associated with increased TNFα and decreased IL-6 production in subcutaneous mammary and lung tumors (15). In a mammary tumor model, increased production of Il-6 was shown to be responsible for myeloid cell recruitment to tumor microenvironment (11). Likewise, we observed reduced production of Il-6 and increased expression levels of TNFα in monocytic cells from livers of TR2^my^KO mice. High levels of pro-inflammatory chemokines, e.g. CCL2 and CCL5, in circulation are associated with poor prognosis for cancer patients (32, 33). Particularly, CCL2 is an apparent key immunosuppressive cytokine in tumors (34). We observed a strong shift in polarization of monocytic myeloid cells in TR2^my^KO mice, associated with reduced expression of CCL2, CCL3, and CCL5 that was confirmed on both transcription and protein levels.

Attenuation of lung metastasis observed in a mammary mouse model of *Tgfbr2*-deficient myeloid cells was associated with decreased production of type II cytokines, Tgfβ1 and Ccl2 (16, 22). We observed similar reduction in Ccl2 amounts in metastatic lungs. Interestingly, reduced amounts of Ccl11, Il-4 and Il-5 were also detected, which is in agreement with reduced alternative polarization of immune cells. Myeloid cell recruitment to the lungs during mammary carcinoma metastasis resulted in a decreased IFN-γ production and increase proinflammatory cytokines (13). The gene expression analysis of sorted monocytes (Ly6C^+^) and neutrophils (Ly6G^+^) showed small differences in inflammatory genes between TR2^my^KO and Ctrl mice. Finally, we provide evidence that spontaneous lung metastasis of LLC1.1 cells is controlled by CD8^+^ T cells in TR2^my^KO mice.

The functional Tgfβ signaling in myeloid cells promotes lung metastasis of mammary tumors through immunosuppression (16, 22). We detected no significant difference in Tgfβ levels in metastatic lungs nor livers between TR2^my^KO and Ctrl mice. However, the increased Tgfβ amounts during early metastasis suggests that Tgfβ affects this phase of the metastatic process. Both tumor cell lines, LLC1.1 and MC-38, produce significant amounts of Tgfβ1 (**Supplementary Figure 7**). Interestingly, MC-38 tumor cells deficient in Tgfβ1 production produced no experimental metastasis irrespective of target tissue (data not shown). Thus, tumor-derived Tgfβ appears to be essential during the early stages of metastasis. How other cells in the metastatic microenvironment, such as fibroblasts, contribute to Tgfβ levels and thereby metastasis requires further investigation.

Taken together, these data indicate that TGFβ modulates monocytic myeloid cells, and thereby promotes metastasis both in the lungs and the liver. While the function of myeloid cells during lung metastasis results in suppression of CD8^+^ T cells, myeloid cells seems to contribute directly to liver metastasis. One caveat of this study is the use of the experimental liver metastasis model. Nevertheless, we observed pronounced differences in myeloid cell infiltration in liver metastatic foci, which was dependent on a functional Tgfβ-signaling in myeloid cells. Recently, the inefficacy of immunotherapy on liver metastasis was linked to the macrophage-assisted elimination of T cells (35), which indicates an involvement of macrophages in liver metastasis. Our findings demonstrate that monocytic myeloid cell infiltration of the liver directly contributes to tumor growth during liver metastasis. Further studies will show whether targeting of TGFβ-signaling during metastasis may lead to therapeutic application in cancer patients.

## Data Availability Statement

The original contributions presented in the study are included in the article/**Supplementary Material**. Further inquiries can be directed to the corresponding author.

## Ethics Statement

The animal study was reviewed and approved by Veterinary Office of Kanton Zurich.

## Author Contributions

Conception and Design, LB and MH. Methodology, CS, MR, and MV. Data acquisition, CS, MV, and MR. Analysis and interpretation of data, CS, MV, MR, and LB. Writing/editing of manuscript, CS, MV, MR, MH, and LB. All authors contributed to the article and approved the submitted version.

## Funding

This study was supported by the SNF grant #310030-173076 (LB) and by a research credit of the University of Zurich #K-41406-01 (MR). MH was supported by the SFB TR209 and an ERC Consolidator grant (HepatometaboPath).

## Conflict of Interest

The authors declare that the research was conducted in the absence of any commercial or financial relationships that could be construed as a potential conflict of interest.

## Publisher’s Note

All claims expressed in this article are solely those of the authors and do not necessarily represent those of their affiliated organizations, or those of the publisher, the editors and the reviewers. Any product that may be evaluated in this article, or claim that may be made by its manufacturer, is not guaranteed or endorsed by the publisher.

## References

[1] Salmon H Remark R Gnjatic S Merad M. Host Tissue Determinants of Tumour Immunity. Nat Rev Cancer (2019) 19(4):215–27. doi: 10.1038/s41568-019-0125-9 PMC7787168 30867580

[2] Quail DF Joyce JA. Microenvironmental Regulation of Tumor Progression and Metastasis. Nat Med (2013) 19(11):1423–37. doi: 10.1038/nm.3394 PMC3954707 24202395

[3] Pickup M Novitskiy S Moses HL. The Roles of TGFbeta in the Tumour Microenvironment. Nat Rev Cancer (2013) 13(11):788–99. doi: 10.1038/nrc3603 PMC4025940 24132110

[4] Kubiczkova L Sedlarikova L Hajek R Sevcikova S. TGF-Beta - An Excellent Servant But a Bad Master. J Transl Med (2012) 10:183. doi: 10.1186/1479-5876-10-183 22943793 PMC3494542

[5] Batlle E Massague J. Transforming Growth Factor-Beta Signaling in Immunity and Cancer. Immunity (2019) 50(4):924–40. doi: 10.1016/j.immuni.2019.03.024 PMC7507121 30995507

[6] de Kruijf EM Dekker TJ Hawinkels LJ Putter H Smit VT Kroep JR. The Prognostic Role of TGF-Beta Signaling Pathway in Breast Cancer Patients. Ann Oncol (2013) 24(2):384–90. doi: 10.1093/annonc/mds333 23022998

[7] Flavell RA Sanjabi S Wrzesinski SH Licona-Limon P. The Polarization of Immune Cells in the Tumour Environment by TGFbeta. Nat Rev Immunol (2010) 10(8):554–67. doi: 10.1038/nri2808 PMC3885992 20616810

[8] Tauriello DVF Palomo-Ponce S Stork D Berenguer-Llergo A Badia-Ramentol J Iglesias M. TGFbeta Drives Immune Evasion in Genetically Reconstituted Colon Cancer Metastasis. Nature (2018) 554(7693):538–43. doi: 10.1038/nature25492 29443964

[9] Gabrilovich DI Nagaraj S. Myeloid-Derived Suppressor Cells as Regulators of the Immune System. Nat Rev Immunol (2009) 9(3):162–74. doi: 10.1038/nri2506 PMC2828349 19197294

[10] Wang D Sun H Wei J Cen B DuBois RN. CXCL1 Is Critical for Premetastatic Niche Formation and Metastasis in Colorectal Cancer. Cancer Res (2017) 77(13):3655–65. doi: 10.1158/0008-5472.CAN-16-3199 PMC5877403 28455419

[11] Oh K Lee OY Shon SY Nam O Ryu PM Seo MW. A Mutual Activation Loop Between Breast Cancer Cells and Myeloid-Derived Suppressor Cells Facilitates Spontaneous Metastasis Through IL-6 Trans-Signaling in a Murine Model. Breast Cancer Res (2013) 15(5):R79. doi: 10.1186/bcr3473 24021059 PMC3979084

[12] Lesokhin AM Hohl TM Kitano S Cortez C Hirschhorn-Cymerman D Avogadri F. Monocytic CCR2(+) Myeloid-Derived Suppressor Cells Promote Immune Escape by Limiting Activated CD8 T-Cell Infiltration Into the Tumor Microenvironment. Cancer Res (2012) 72(4):876–86. doi: 10.1158/0008-5472.CAN-11-1792 PMC3288305 22174368

[13] Yan HH Pickup M Pang Y Gorska AE Li Z Chytil A. Gr-1+CD11b+ Myeloid Cells Tip the Balance of Immune Protection to Tumor Promotion in the Premetastatic Lung. Cancer Res (2010) 70(15):6139–49. doi: 10.1158/0008-5472.CAN-10-0706 PMC4675145 20631080

[14] Fridlender ZG Sun J Kim S Kapoor V Cheng G Ling L. Polarization of Tumor-Associated Neutrophil Phenotype by TGF-Beta: “N1” Versus “N2” TAN. Cancer Cell (2009) 16(3):183–94. doi: 10.1016/j.ccr.2009.06.017 PMC2754404 19732719

[15] Novitskiy SV Pickup MW Chytil A Polosukhina D Owens P Moses HL. Deletion of TGF-Beta Signaling in Myeloid Cells Enhances Their Anti-Tumorigenic Properties. J Leukoc Biol (2012) 92(3):641–51. doi: 10.1189/jlb.1211639 PMC3427612 22685318

[16] Pang Y Gara SK Achyut BR Li Z Yan HH Day CP. TGF-Beta Signaling in Myeloid Cells Is Required for Tumor Metastasis. Cancer Discov (2013) 3(8):936–51. doi: 10.1158/2159-8290.CD-12-0527 PMC4678771 23661553

[17] Yan HH Jiang J Pang Y Achyut BR Lizardo M Liang X. CCL9 Induced by TGFbeta Signaling in Myeloid Cells Enhances Tumor Cell Survival in the Premetastatic Organ. Cancer Res (2015) 75(24):5283–98. doi: 10.1158/0008-5472.CAN-15-2282-T PMC5120555 26483204

[18] Borsig L Wong R Hynes RO Varki NM Varki A. Synergistic Effects of L- and P-Selectin in Facilitating Tumor Metastasis can Involve Non-Mucin Ligands and Implicate Leukocytes as Enhancers of Metastasis. Proc Natl Acad Sci USA (2002) 99(4):2193–8. doi: 10.1073/pnas.261704098 PMC122341 11854515

[19] Hauselmann I Roblek M Protsyuk D Huck V Knopfova L Grassle S. Monocyte Induction of E-Selectin-Mediated Endothelial Activation Releases VE-Cadherin Junctions to Promote Tumor Cell Extravasation in the Metastasis Cascade. Cancer Res (2016) 76(18):5302–12. doi: 10.1158/0008-5472.CAN-16-0784 PMC6322653 27488527

[20] Gabrilovich DI Ostrand-Rosenberg S Bronte V. Coordinated Regulation of Myeloid Cells by Tumours. Nat Rev Immunol (2012) 12(4):253–68. doi: 10.1038/nri3175 PMC3587148 22437938

[21] Ferjancic S Gil-Bernabe AM Hill SA Allen PD Richardson P Sparey T. VCAM-1 and VAP-1 Recruit Myeloid Cells That Promote Pulmonary Metastasis in Mice. Blood (2013) 121(16):3289–97. doi: 10.1182/blood-2012-08-449819 23407548

[22] Yang L Huang J Ren X Gorska AE Chytil A Aakre M. Abrogation of TGF Beta Signaling in Mammary Carcinomas Recruits Gr-1+CD11b+ Myeloid Cells That Promote Metastasis. Cancer Cell (2008) 13(1):23–35. doi: 10.1016/j.ccr.2007.12.004 18167337 PMC2245859

[23] Wolf MJ Hoos A Bauer J Boettcher S Knust M Weber A. Endothelial CCR2 Signaling Induced by Colon Carcinoma Cells Enables Extravasation *via* the JAK2-Stat5 and P38mapk Pathway. Cancer Cell (2012) 22(1):91–105. doi: 10.1016/j.ccr.2012.05.023 22789541

[24] Läubli H Stevenson JL Varki A Varki NM Borsig L. L-Selectin Facilitation of Metastasis Involves Temporal Induction of Fut7-Dependent Ligands at Sites of Tumor Cell Arrest. Cancer Res (2006) 66(3):1536–42. doi: 10.1158/0008-5472.CAN-05-3121 16452210

[25] Coffelt SB Wellenstein MD de Visser KE. Neutrophils in Cancer: Neutral No More. Nat Rev Cancer (2016) 16(7):431–46. doi: 10.1038/nrc.2016.52 27282249

[26] Lewis CE Pollard JW. Distinct Role of Macrophages in Different Tumor Microenvironments. Cancer Res (2006) 66(2):605–12. doi: 10.1158/0008-5472.CAN-05-4005 16423985

[27] Kratochvill F Neale G Haverkamp JM Van de Velde LA Smith AM Kawauchi D. TNF Counterbalances the Emergence of M2 Tumor Macrophages. Cell Rep (2015) 12(11):1902–14. doi: 10.1016/j.celrep.2015.08.033 PMC4581986 26365184

[28] Shaul ME Levy L Sun J Mishalian I Singhal S Kapoor V. Tumor-Associated Neutrophils Display a Distinct N1 Profile Following TGFbeta Modulation: A Transcriptomics Analysis of Pro- vs. Antitumor TANs. Oncoimmunology (2016) 5(11):e1232221. doi: 10.1080/2162402X.2016.1232221 27999744 PMC5139653

[29] Dranoff G. Cytokines in Cancer Pathogenesis and Cancer Therapy. Nat Rev Cancer (2004) 4(1):11–22. doi: 10.1038/nrc1252 14708024

[30] Kitamura T Qian BZ Pollard JW. Immune Cell Promotion of Metastasis. Nat Rev Immunol (2015) 15(2):73–86. doi: 10.1038/nri3789 25614318 PMC4470277

[31] Kalluri R. The Biology and Function of Fibroblasts in Cancer. Nat Rev Cancer (2016) 16(9):582–98. doi: 10.1038/nrc.2016.73 27550820

[32] Nagarsheth N Wicha MS Zou W. Chemokines in the Cancer Microenvironment and Their Relevance in Cancer Immunotherapy. Nat Rev Immunol (2017) 17(9):559–72. doi: 10.1038/nri.2017.49 PMC5731833 28555670

[33] Qian BZ Li J Zhang H Kitamura T Zhang J Campion LR. CCL2 Recruits Inflammatory Monocytes to Facilitate Breast-Tumour Metastasis. Nature (2011) 475(7355):222–5. doi: 10.1038/nature10138 PMC3208506 21654748

[34] Fridlender ZG Buchlis G Kapoor V Cheng G Sun J Singhal S. CCL2 Blockade Augments Cancer Immunotherapy. Cancer Res (2010) 70(1):109–18. doi: 10.1158/0008-5472.CAN-09-2326 PMC2821565 20028856

[35] Yu J Green MD Li S Sun Y Journey SN Choi JE. Liver Metastasis Restrains Immunotherapy Efficacy *via* Macrophage-Mediated T Cell Elimination. Nat Med (2021) 27(1):152–64. doi: 10.1038/s41591-020-1131-x PMC8095049 33398162

