# Supplementary material for: TGFβ Signaling in Myeloid Cells Promotes Lung and Liver Metastasis Through Different Mechanisms
Source: Front Oncol. 2021 Nov 18;11:765151. doi: 10.3389/fonc.2021.765151 (PMC8637420; doi:10.3389/fonc.2021.765151)
Supplement: Supplementary file 1 [file DataSheet_1.pdf]

Supplementary Data

Stefanescu C et al. **TGFβ signaling in myeloid cells promotes lung and liver metastasis through different mechanisms.**

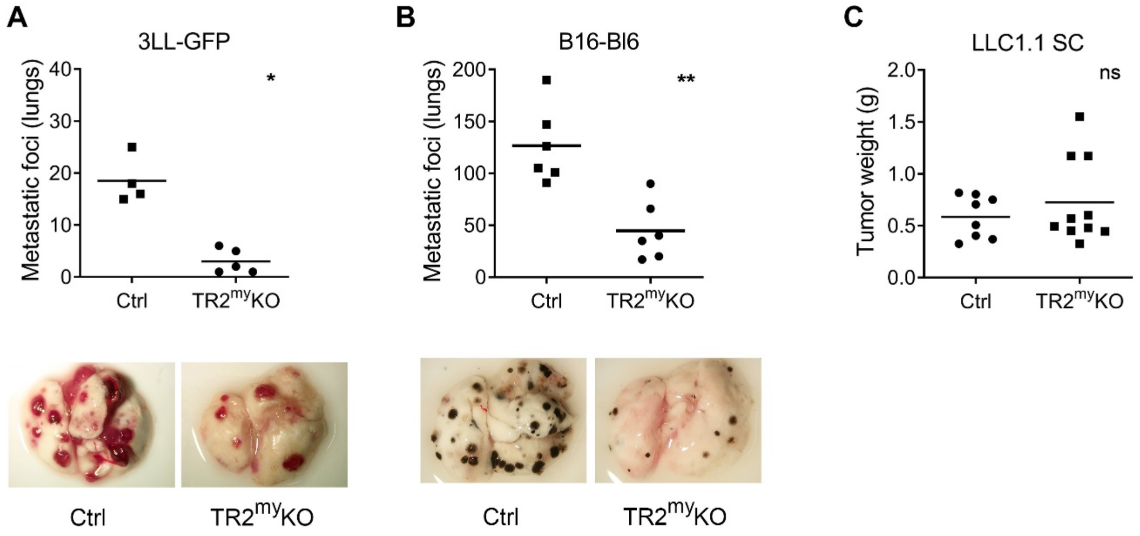

Supplementary Figure 1. Experimental lung metastasis and tumor growth in mice with myeloid TGFβRII deficiency. LysMCre^{+}/TGFβRII$^{fl/fl}$ myeloid deficient TGFβ signaling mice (TR2^{my}KO) and control mice LysMCre^{neg}/TGFβRII$^{fl/fl}$ (Ctrl). **A)** Macroscopic evaluation of lung metastasis 14 days after intravenous injection of lung cancer cell line 3LL-GFP (150'000 cells). **B)** Macroscopic evaluation of lung metastasis 14 days after intravenous injection of melanoma cell line B16-BL6 (150'000 cells). **C)** Primary tumor weight of LLC1.1 subcutaneous tumors at day 18, time of resection. Statistical significance was assessed using the Mann-Whitney test; *, $p < 0.05$; **, $p < 0.01$; ns, not significant.

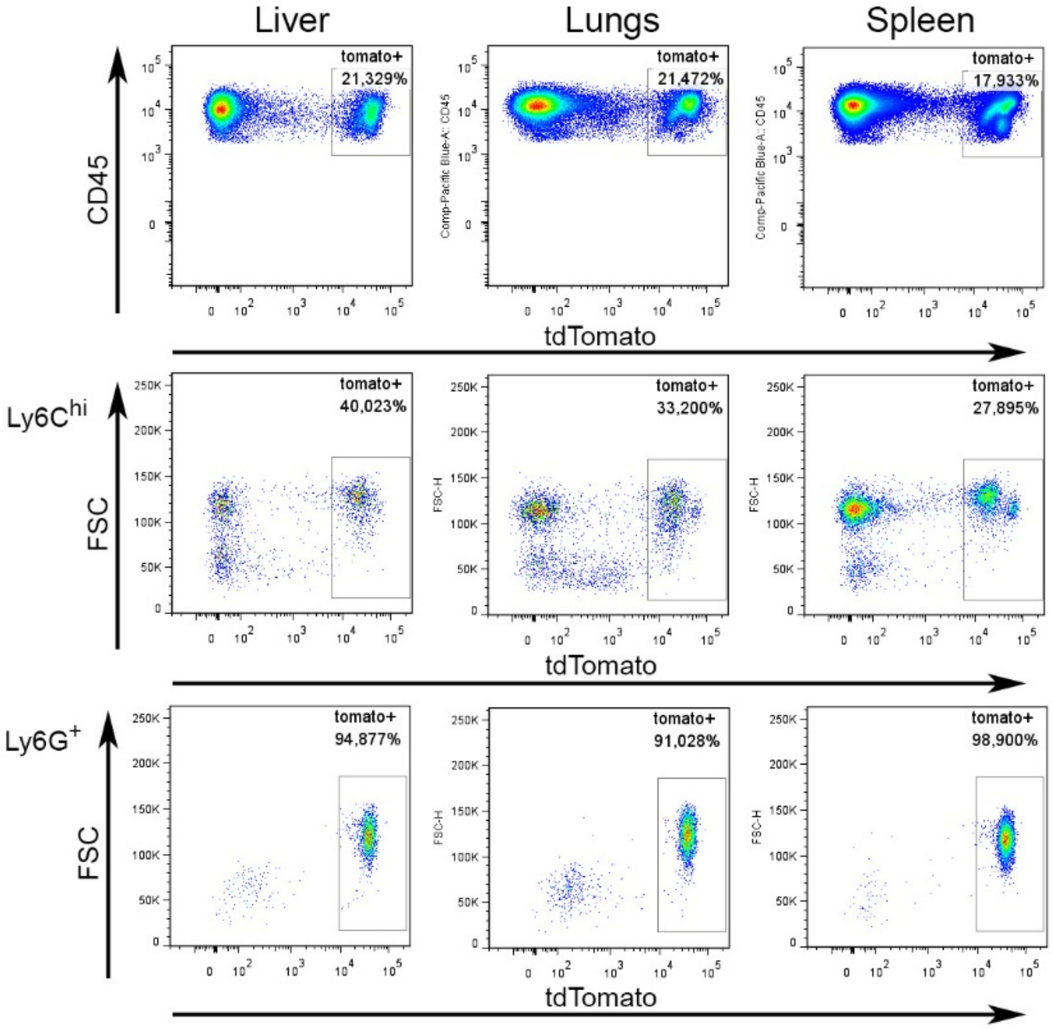

Supplementary Figure 2. Analysis of LysMCre⁺/TGFβRII$^{fl/fl}$/tdT⁺ reporter mice. Analysis of tdTomato-expressing CD45⁺ cells in various organs (top), specifically quantified in Ly6C^{hi} monocytes (middle) and Ly6G⁺ neutrophils (bottom) using flow cytometry

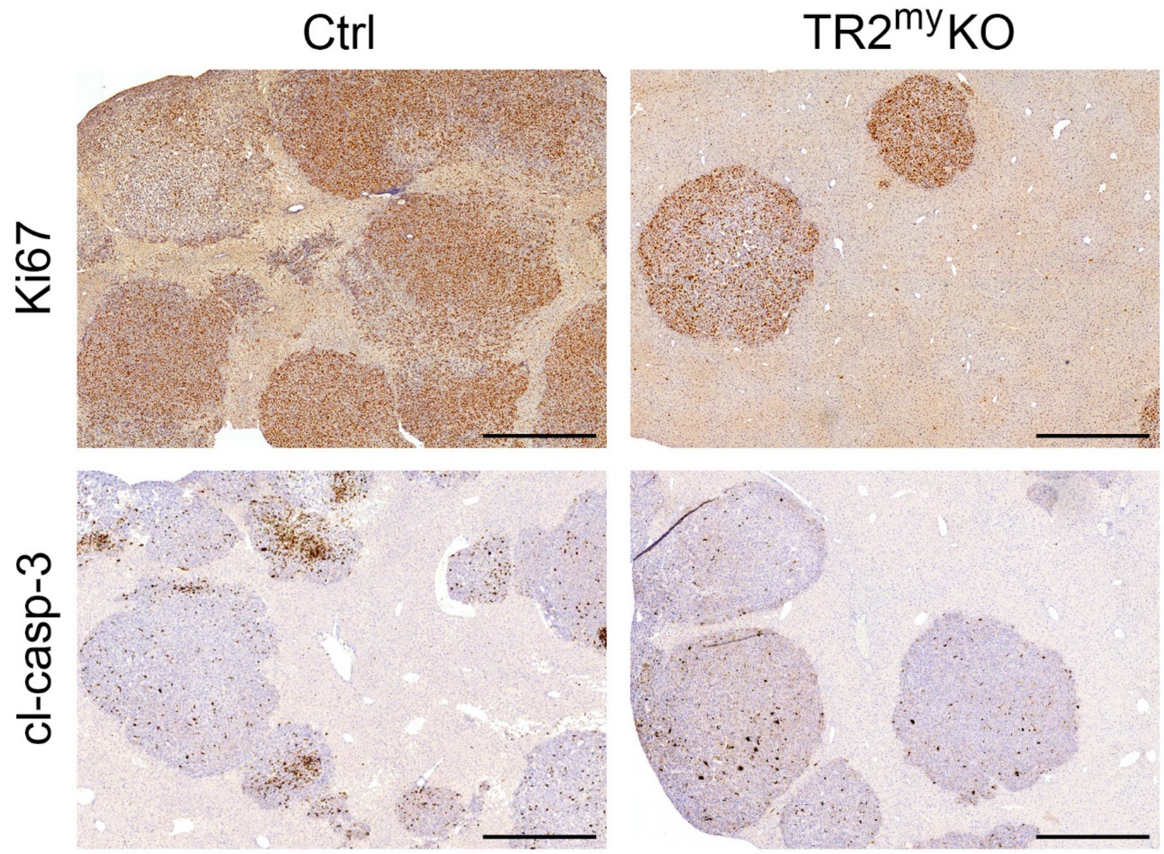

Supplementary Figure 3. Histological evaluation of liver metastasis in Ctrl and TR2myKO mice. Representative images of livers from mice terminated at day 28 post-tumor cell injection. Tissue sections (5 μm) were stained with cl-Casp3, and Ki67 Abs (brown), and counterstained with hematoxylin. Bar = 1 mm.

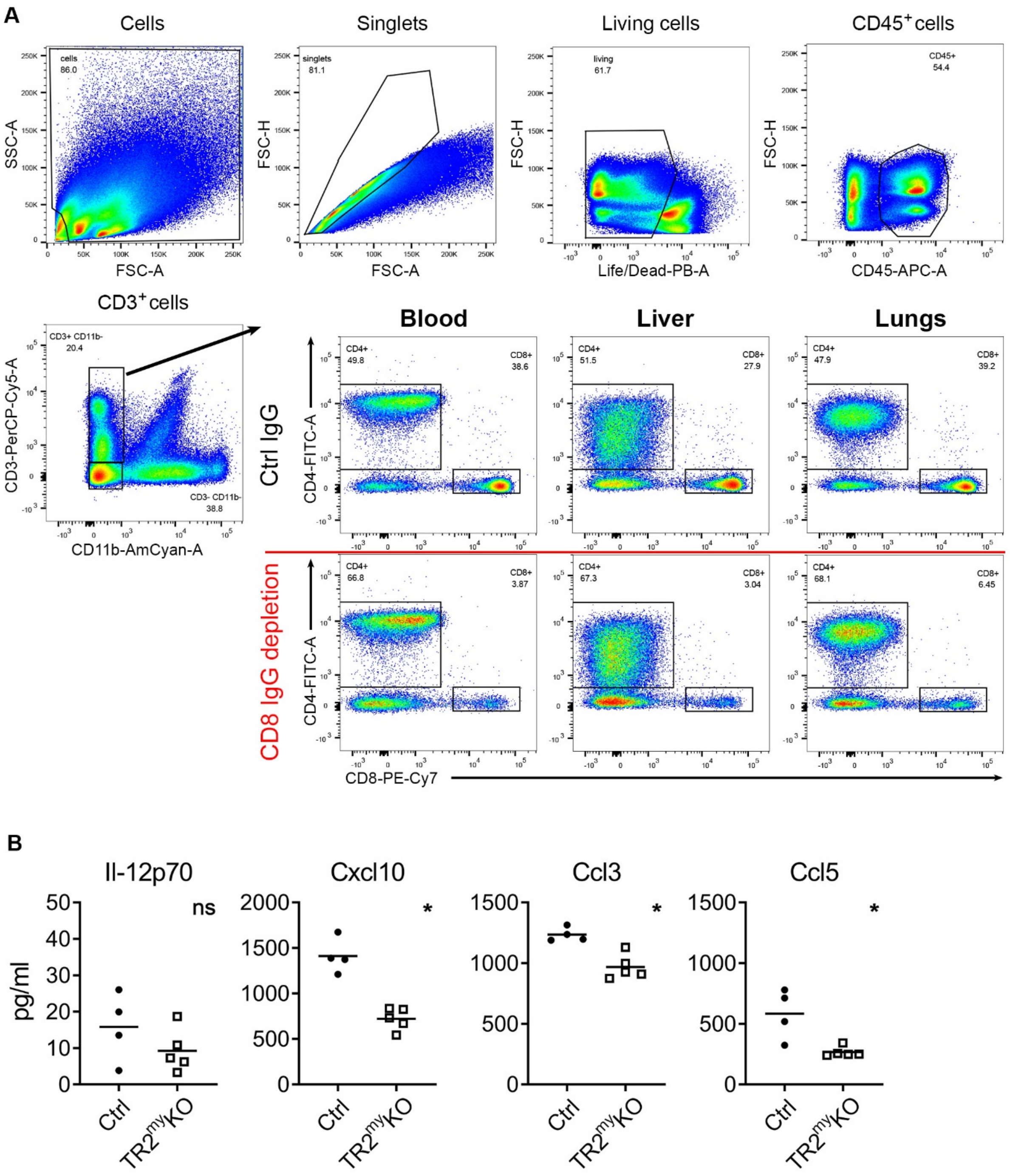

Supplementary Figure 4. Depletion of CD8 cells and analysis of myeloid cells from metastatic livers. A) Gating strategy and the analysis of CD8 depletion in mice 48 h after intraperitoneal injection with 15 μg of anti-CD8 antibody (clone 2.43; BioXCell) compared to mice injected with 15 μg of the control IgG (clone 2A3, BioXCell). The presence of CD8 cells in the circulation (blood), and in the perfused liver and lungs were quantified. **B)** Analysis of cytokine production by sorted tdT^{+} Ly6C^{hi} monocytes from livers of Ctrl and TR2^{my}KO mice 14 days post-intrasplenic injection of MC-38GFP cells. Cells were stimulated overnight and secreted cytokines were measured in supernatants. Statistical significance was assessed using Mann-Whitney test: ns, not significant; *, $p<0.05$.

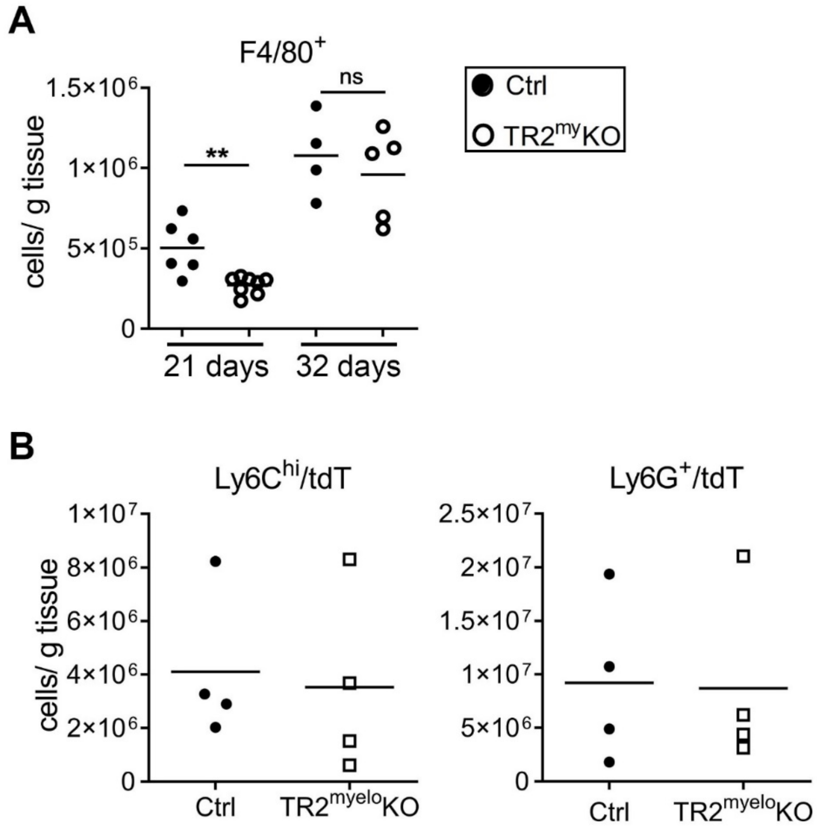

Supplementary Figure 5. Analysis of myeloid cell recruitment to the metastatic lungs in myeloid cell-Tgfb2-deficient mice. A) Flow cytometry analysis of the F4/80^{+} macrophages in lungs at day 21 and 32 after subcutaneous injection of LLC1.1 cells in TR2^{myelo}KO and Ctrl mice, respectively. **B)** Analysis of tdTomato reporter expression in TR2^{my}KO (LysMCre^{+}/Tgfbr2$^{fl/fl}$/tdT^{+}) and Ctrl mice (LysMCre^{+}/Tgfbr2^{wt}/tdT^{+}). Flow cytometry analysis of tdT-positive Ly6C^{hi} and Ly6G^{+} myeloid cells in perfused lungs from TR2^{my}KO and Ctrl mice at day 32 post-subcutaneous injection of LLC1.1 cells.

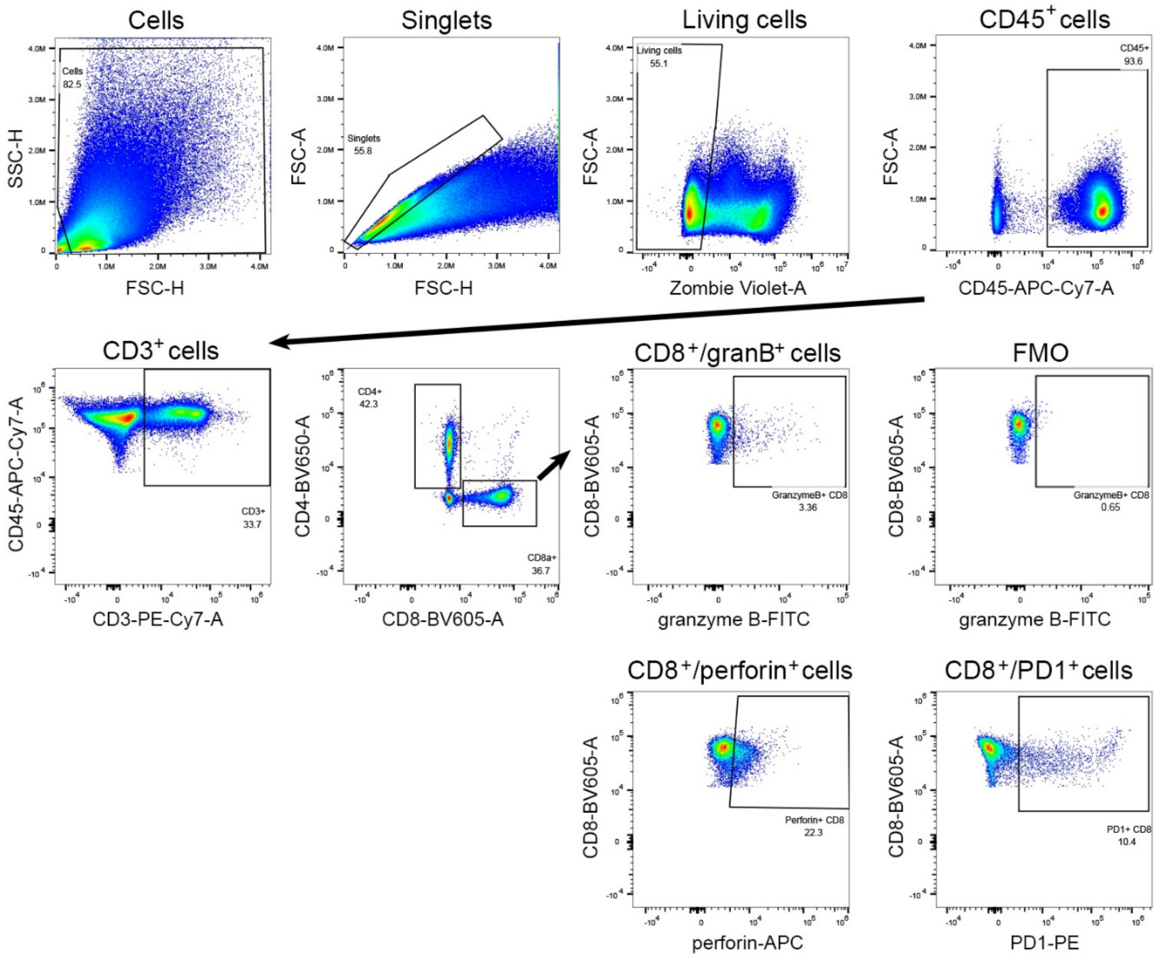

Supplementary Figure 6. Flow cytometry analysis of effector CD8 cells from lung metastasis in Ctrl and TR2^{my}KO mice at day 32. Gating strategy for CD8 effector cells stained with granzyme B, perforin and PD1 antibodies, respectively, for the results shown in Figure 6E.

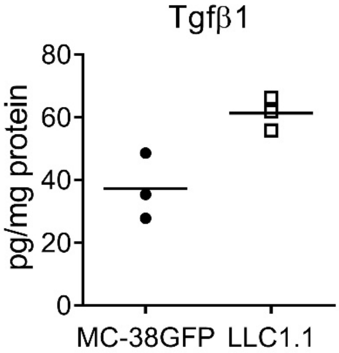

Supplementary Figure 7. Determination of Tgfβ1 production by tumor cells. MC-38GFP and LLC1.1 cells were grown to 60% confluency, medium was changed to serum reduced DMEM with 2% FBS and the supernatant was collected after 3 days. Tgfβ1 concentration from three independent experiments was determined using TGFβ Platinum Elisa (Thermo Fisher) and presented in total protein amount in the supernatant.

Supplementary Table S1

Ccl3	Fw 5`-ATG GAG CTG ACA CCC CGA CT-3`;<br>Rv 5`-GGT CTC TTT GGA GTC AGC GCA-3`	
ICAM1	Fw 5`-CCC CGC AGG TCC AAT TCA CA-3`;<br>Rv 5`-CCA AGC AGT CCG TCT CGT CC-3`	
Tnfα	Fw 5`-GTG ATC GGT CCC CAA AGG GAT G-3`;<br>Rv 5`-ACT TGG TGG TTT GCT ACG ACG TG-3`	
Ccl5	Fw 5`-GCTGCTTTGCCTACCTCTCC-3`; Rv 5`-TCGAGTGACAAACACGACTGC-3`	
MMP9	Fw 5`-CGGACATTGTCATCCAGTTT-3`; Rv 5`-GGGATCACGACGCCTTT-3`	
VEGF	Fw 5`- CTTGTTCAGAGCGGAGAAAGC-3`;<br>Rv 5`- ACATCTGCAAGTACGTTCGTT-3`	(1)
Ccl2	Fw 5`-TTA ACG CCC CAC TCA CCT GC-3`;<br>Rv 5`-TGG GGT CAG CAC AGA CCT CTC-3`	
Arg1	Fw 5`-AGC ATG AGC TCC AAG CCA AAG T-3`;<br>Rv 5`-GTG GTC TCT CAC GTC ATA CTC TGT T-3`	
IL-1b	Fw 5`-TCC AGG ATG AGG ACA TGA GCA C-3`;<br>Rv 5`-GAA CGT CAC ACA CCA GCA GGT TA-3`	
SOCS3	Fw 5`-GCTGGCCAAAGAAATAACCA-3`;<br>Rv 5`- AGCTCACCAGCCTCATCTGT-3`	(2)
Clec7a	Fw 5`-ATCAGCATTCTTCCCCAACTCG-3`;<br>Rv 5`- CAGTTCCTTCTCACAGATACTGTATGA-3`	(3)
Chi3l3	Fw 5`- CTGAATGAAGGAGCCACTGA-3`;<br>Rv 5`- AGCCACTGAGCCTTCAACTT-3`	(4)

References

1. Fridlender ZG, Sun J, Kim S, Kapoor V, Cheng G, Ling L, et al. Polarization of tumor-associated neutrophil phenotype by TGF-beta: "N1" versus "N2" TAN. Cancer Cell. 2009;16:183-94.
2. Swiderski K, Thakur SS, Naim T, Trieu J, Chee A, Stapleton DI, et al. Muscle-specific deletion of SOCS3 increases the early inflammatory response but does not affect regeneration after myotoxic injury. Skelet Muscle. 2016;6:36.

3. Viriyakosol S, Jimenez Mdel P, Gurney MA, Ashbaugh ME, Fierer J. Dectin-1 is required for resistance to coccidioidomycosis in mice. MBio. 2013;4:e00597-12.
4. Wan S, Sun X, Wu F, Yu Z, Wang L, Lin D, et al. Chi3l3: a potential key orchestrator of eosinophil recruitment in meningitis induced by Angiostrongylus cantonensis. J Neuroinflammation. 2018;15:31.
